# Immune-related adverse events and anti-tumor efficacy of immune checkpoint inhibitors

**DOI:** 10.1186/s40425-019-0805-8

**Published:** 2019-11-15

**Authors:** Satya Das, Douglas B. Johnson

**Affiliations:** Vanderbilt University Medical Center, Department of Medicine, Division of Hematology and Oncology, 1301 Medical Center Drive, Nashville, 37232 USA

**Keywords:** Immune-related adverse events, Immune checkpoint inhibitor efficacy, Autoimmunity and anti-tumor effect, Anti-programmed cell death protein 1, Anti-programmed death-ligand 1, Anti-cytotoxic T-lymphocyte-associated protein 4

## Abstract

Although immune checkpoint inhibitors (ICIs) have transformed the treatment landscape for patients with many advanced malignancies, only 15–60% of patients respond, leaving a broad swath of patients who do not derive benefit. Identifying biomarkers to optimally identify patients who will benefit from ICIs is a major research focus for the oncology community. Thus far, predictive biomarker research has focused on tumor signatures such as microsatellite instability, programmed death-ligand 1 (PD-L1) expression and tumor mutational burden; clinical biomarkers have been far less studied. One potential clinical biomarker for ICI response in patients is immune-related adverse event (IRAE) onset.

IRAEs are thought to represent bystander effects from activated T-cells and it is plausible that patients responding to ICIs would have greater likelihood of autoimmune toxicities (e.g. due to a more competent/treatment-responsive immune system, or cross-reactivity between tumor and host tissue). Earlier studies in melanoma patients however, suggested no association between IRAE onset and anti-cytotoxic T-lymphocyte-associated protein 4 (CTLA-4) antibody efficacy. In contrast, a growing body of literature suggests IRAE onset is predictive of anti-programmed cell death protein 1 (PD-1) and anti-PD-L1 antibody response across a variety of solid tumors. Most of these studies report that patients who experienced IRAEs demonstrate marked improvements in progression-free survival, overall survival and overall response rate compared to those lacking toxicity.

Key questions regarding the association between IRAE onset and ICI efficacy remain. The most pertinent of these involve whether the association is only relevant for patients treated with anti-PD-1 and anti-PD-L1 antibodies and whether IRAE site, severity, timing of onset and management influence ICI efficacy. Herein, we discuss the seminal studies which have begun to address these questions and have shaped the narrative about the predictive value of IRAE onset for patients on ICIs, in this review.

## Introduction

Immune checkpoint inhibitors (ICIs) have transformed the treatment landscape for patients with advanced malignancies. Programmed cell death protein 1 (PD-1), programmed death-ligand 1 (PD-L1) and cytotoxic T-lymphocyte-associated protein 4 (CTLA-4) are checkpoints which have been successfully targeted with antagonist antibodies. Over the last several years, ICIs have garnered first- and later-line FDA approvals in non-small cell lung cancer (NSCLC), renal cell carcinoma (RCC), urothelial carcinoma (UCC), melanoma, classical Hodgkin’s lymphoma, microsatellite instability-high (MSI-H) tumors, hepatocellular carcinoma (HCC), gastric and gastroesophageal junction (GA & GEJ) adenocarcinoma, merkel-cell carcinoma, head and neck squamous cell carcinoma (HNSCC) and others [1–10]. Response rates range from 15 to 30% (in most solid tumors) to 45–60% (in melanoma and MSI-H tumors). However, a large proportion of patients do not respond to these therapies, creating a need to identify biomarkers to predict which patients derive the most benefit from treatment. Predictive biomarker research has predominantly been focused on tumor signatures such as MSI-H status, tumor mutational burden (TMB) and PD-L1 expression [11, 12]; clinical biomarkers, including early-on-treatment pharmacodynamic markers, have been much less studied.

Immune-related adverse event (IRAE) onset may represent one such clinical biomarker for ICI response. Across disease sites, patients who experience IRAEs while on therapy with anti-PD-1 and anti-PD-L1 antibodies have been documented to experience improved outcomes as measured by overall response rate (ORR), progression-free survival (PFS) and overall survival (OS) [13–20]. In patients treated with anti-CTLA-4 antibodies, this association has been less uniform [21–25]. Key questions regarding the complete nature of the relationship between IRAEs and ICI efficacy remain unsettled. The most pertinent of these involve whether IRAE site, severity, timing of onset and management influence ICI effectiveness. We will discuss the seminal studies which have addressed some of these questions and have shaped the narrative about the predictive value of IRAE onset for patients on ICIs in this review. The review will focus on studies in patient populations with FDA-approved indications for ICI therapy, as well as those which include ICIs alone (no chemotherapy combinations), in order to make our conclusions as generalizable as possible.

### Potential mechanism between IRAE onset and anti-tumor effect

Although the precise mechanisms by which IRAEs occur have not been fully uncovered, they are thought to represent bystander effects from activated T-cells and are consistent with the mechanism of action of ICIs [26, 27]. Specifically, tumors inflamed with cytotoxic T lymphocytes prior to treatment then experience further inflammation and tumor-cell death when treated with ICIs. Similarly, an organ with subclinical inflammation may experience pronounced, clinically apparent inflammation when these key negative regulators of T-cell function are removed. However, the mechanisms why specific toxicities occur in specific patients, and the link between toxicity and response, are not yet apparent.

Early studies have begun to address these molecular mechanisms. One set of studies suggests that perhaps IRAEs are triggered by antigens that are common to both tumor and inflamed organ. Under this model, unleashed T cells would target both tissues, producing both toxicity and response. In a post-mortem study of two metastatic melanoma patients who developed fulminant myocarditis after nivolumab plus ipilimumab, infiltrating T-cells and macrophages were found in the myocardial tissue and the cardiac conduction system [28]. Deeper interrogation of the infiltrating T-cells through T-cell receptor (TCR) sequencing revealed common high-frequency TCRs in cardiac muscle, skeletal muscle and tumor. In a recent prospective cohort study of 73 NSCLC patients treated with anti-PD-1 antibodies, 34.2% of patients developed dermatologic IRAEs [29]. TCR clonotype analysis was performed on samples from 4 patients with matched skin and tumor biopsies, revealing that shared T-cell clones between skin and tumor were present in all patients. Subsequent experiments revealed 9 candidate shared antigens between skin and tumor which were successfully able to elicit interferon gamma-based T-cell responses in stimulated peripheral blood mononuclear cells from patients with dermatologic IRAEs.

Other studies which point to the link between T-cells and IRAEs focus on the gut microbiome. Significant differences in microbial diversity and composition have been noted between responding and non-responding melanoma patients treated with anti-PD-1 therapy; different studies suggest different species may be enriched in responding versus non-responding patients [30, 31]. Fecal microbiome transplant experiments in mice models from several of these studies have revealed mechanistic insights. Mice that were transplanted with stool from patients responding to anti-PD-1 antibodies had higher levels of CD8 T-cell density in tumor tissue. Furthermore, mice transplanted with stool from responding patients also had higher levels of CD8 T-cell concentrations in the gut than those transplanted with stool from non-responders. A study of 26 metastatic melanoma patients treated with ipilimumab suggested that patients with baseline gut microbiota enriched with the Faecalibacterium (and other members of the Firmicutes phylum) had improved PFS, OS and higher rates of ICI-induced colitis compared to patients who were not enriched [32]. Patients who were enriched with Firmicutes had a lower proportion of regulatory T-cells and alpha 4 beta 7 integrin positive CD4 and CD8 T-cells than patients who were not enriched. Thus, microbiome composition may be linked with both toxicities and response, although it remains far from clear the importance of various microbial species; further prospective studies are needed.

Other studies suggest there may be mechanisms of autoimmune toxicity which are independent of anti-tumor response. In a model of hypophysitis associated with ipilimumab, SJL mice were treated with an IgG1 hamster antibody blocking CTLA-4, using a dose regimen comparable to the one utilized in humans [33]. Mice treated with the anti-CTLA-4 antibody developed a distinct lymphocytic infiltrate in the pituitary gland. No infiltrate was seen in other organs in treated mice such as the thyroid gland, skin, colon or liver. Pituitary antibodies were not found in pre-treatment mice or in controls. CTLA-4 mRNA expression was detected in the murine pituitary gland, predominantly in lactotrophic and thyrotrophic cells, and was found in much lower levels in the murine thyroid gland. This study suggests pre-existing organ specific antigen expression may be one cause of autoimmune toxicity from ICIs without representing a shared effect from anti-tumor activity.

**Fig. 1:**
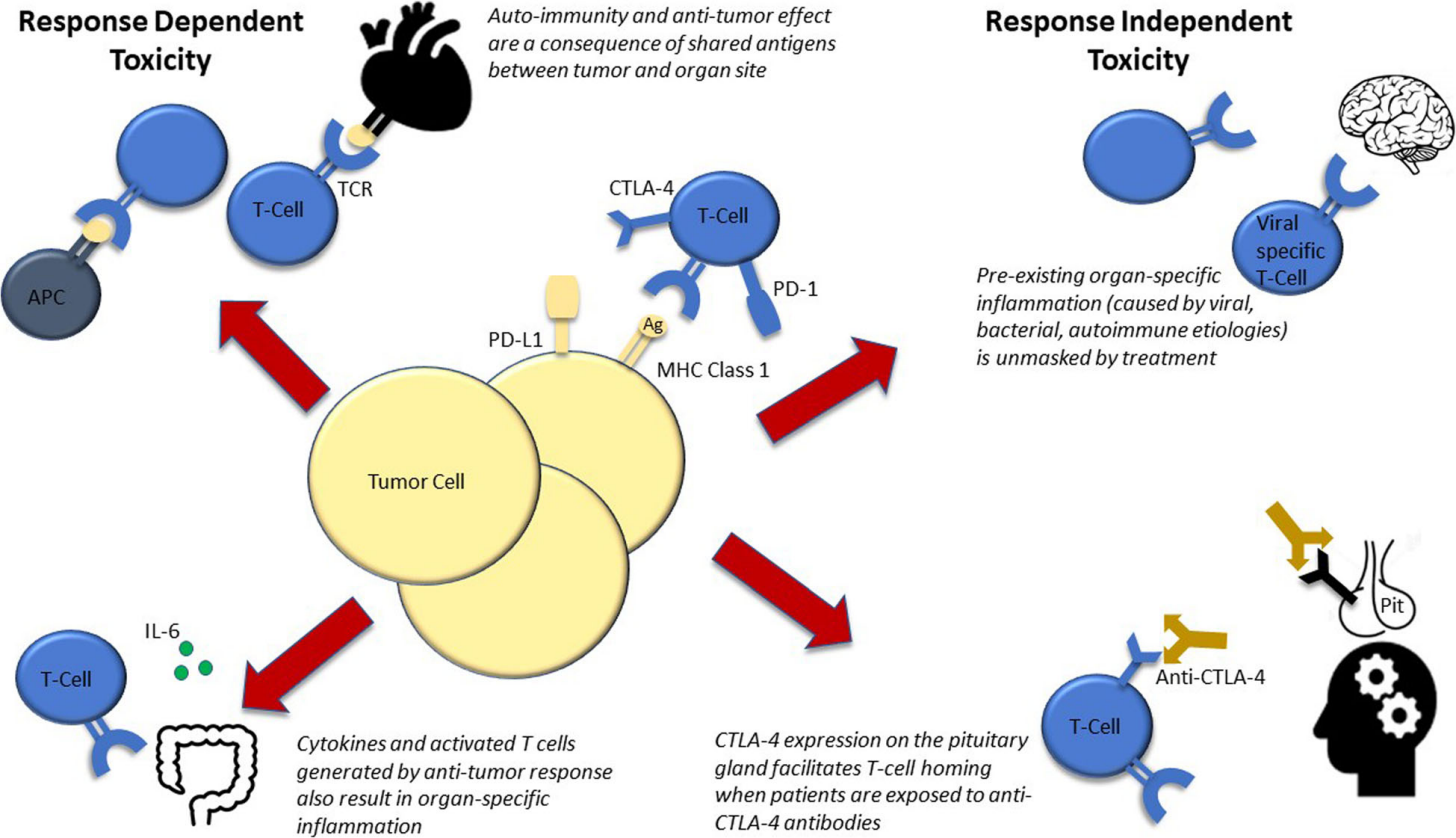
Mechanisms of Response Dependent and Response Independent Autoimmune Toxicity from Immune Checkpoint Inhibitors. On top left is a depiction of myocardial cells expressing shared antigens with the tumor which leads to autoimmunity. On bottom left is a depiction of IL-6 production from T-cell activation resulting in attack on enterocytes. On top right is a depiction of encephalitis as a result of an anti-viral response being unleashed by ICI treatment. On bottom right is a depiction of endogenous CTLA-4 expression on the pituitary gland leading to T-cell attack after anti-CTLA-4 treatment. Abbreviations: APC, antigen presenting cell; TCR, T-cell receptor; CTLA-4, cytotoxic T-lymphocyte-associated protein 4; PD-1, programmed cell death protein 1; PD-L1, programmed death-ligand 1; Ag, antigen; MHC, major histocomptability complex; Pit, pituitary gland

Figure 1 is a representation of anti-tumor response dependent and response independent mechanisms by which autoimmunity may occur in patients treated with ICIs.

### Anti-PD-1 and anti-PD-L1 antibodies

#### NSCLC

First, we will review studies that have assessed efficacy with toxicities globally (as opposed to site-specific toxicities). Both prospective and retrospective analyses in NSCLC patients have demonstrated an association between IRAE onset and efficacy of anti-PD-1 and anti-PD-L1 antibodies. Focusing first on the retrospective studies, in an observational study of 270 largely pretreated patients with metastatic NSCLC, treated with at least one dose of anti-PD-L1 or anti-PD-1 antibodies, outcomes were compared between patients who did and did not experience IRAEs [18]. Most patients (89.3%) received anti-PD-1 while the remainder (10.7%) received anti-PD-L1 antibodies. Of the included patients, 44% experienced any grade IRAEs with the most common sites of involvement being endocrine (20%), dermatologic (7%) and gastrointestinal toxicities (7%). Patients who experienced IRAEs had superior PFS and OS compared to those who did not experience IRAEs (OS: not reached (NR) versus (vs) 8.21 months (hazard ratio (HR) 0.29; 95% confidence interval (CI) 0.18–0.46; *p* = .001); PFS: 5.2 vs 1.97 months (HR 0.42; 95% CI 0.32–0.57; *p* < .001)). ORR (22.9% vs 5.7%, *p* < .0001) and disease control rate (DCR) (76% vs 58%, *p* < .001) were also prolonged in patients who experienced IRAEs compared to those who did not experience them. Among patients who were on ICIs for > 3 months and > 6 months, there were no differences in rates of IRAEs. There were no statistically significant differences in OS, PFS, ORR and DCR in patients based on IRAE grade. When looking at outcomes in patients separated by IRAE type, patients who experienced thyroiditis had statistically significant improvements in OS and PFS compared to patients who did not experience the endocrinopathy (OS: NR vs 18.2 months (HR 0.46; 95% CI 0.25–0.86; *p* = .01); PFS: 8.05 vs 2.59 months (HR 0.58; 95% CI .39–.85; *p* = .005)). There were no significant differences when looking at outcomes in patients by timing of IRAE onset (< 3 months vs ≥ 3 months).

In another large retrospective analysis, outcomes in 195 NSCLC patients from multiple institutions treated with nivolumab who did and did not experience IRAES were assessed [34]. Of the included patients, 43.6% developed IRAEs with the most commonly involved sites being endocrine, gastrointestinal and dermatologic (unspecified percentages). Patient who experienced IRAEs had statistically significant improvements in ORR (43.5% vs 10%, *p* < .001), PFS (5.7 vs 2.0 months (HR 0.41; 95% CI 0.3–0.57; *p* < .001)) and OS (17.8 vs 4.0 months (HR 0.33; 95% CI 0.23–0.47; *p* < .001)) compared to their counterparts who did not experience IRAEs. A 12-week landmark analysis confirmed the same statistically significant differences between patients who did and did not develop IRAEs.

A prospective observational study assessing outcomes by IRAE presence in 38 NSCLC patients treated with nivolumab was reported [19]. Of the included patients, 28.9% experienced an IRAE with a median time to IRAE onset of 50 days. Patients with IRAEs had significantly improved RR (63.6% vs 7.4%, *p* < .01) and PFS (not reached vs 49 days (HR 0.1; 95% CI .02–.37; *p* < .001)) compared to those who did not experience IRAEs. An exploratory analysis comparing PFS in patients with pneumonitis vs those with other IRAEs was performed with no significant differences found between the two groups.

Although only several studies in patients with NSCLC have been highlighted, other studies have demonstrated similar correlation between IRAE onset and ICI efficacy [35, 36].

#### Melanoma

In metastatic melanoma patients, the association between IRAE onset and anti-PD-1 antibody efficacy is not as linear as the relationship seen in other disease types. Though several retrospective analyses suggest improved outcomes in patients based on IRAE presence, not all measured outcomes are uniformly improved in patients with IRAEs. A retrospective analysis of 173 patients with metastatic melanoma treated with anti-PD-1 antibody therapy from a single-center assessed outcomes in patients based upon a variety of factors including IRAE presence [37]. Of patients in the analysis, 59% experienced IRAEs with the most common sites being dermatologic (13%), hepatic (11%) and endocrine (8%). IRAE onset was not significantly associated with ORR in patients (HR 1.95; 95% CI 0.91–4.15; *p* = .082) while was significantly associated with DCR (HR 1.98; 95% CI 1.07–3.67; *p* = .029). It is possible IRAE onset was not significantly associated with ORR given the limitations of ORR as a measure of ICI response in patients [38]. On multivariate analysis, the only factor that was independently associated with PFS was IRAE onset (HR 0.47; 95% CI 0.26–0.86; *p* = .016). With regards to OS, on multivariable analysis, IRAE presence remained significantly associated with the outcome (HR 0.39; 95% CI 0.18–0.81; *p* = .007). Among patients who experienced IRAEs, patients who experienced vitiligo had an improved OS compared to those with all other IRAEs however this was not statistically significant (*p* = .061).

A retrospective analysis analyzed outcomes of 576 melanoma patients pooled from several studies treated with nivolumab [39]. Of the patients, 49% experienced IRAEs with the most common IRAE sites being dermatologic (34%), gastrointestinal (13.4%) and endocrine (7.8%). In a multivariate analysis which adjusted for doses of nivolumab received, tumor PD-L1 level and baseline lactate dehydrogenase levels, ORR was significantly better in patients who experienced any-grade IRAEs than those who did not experience them (48.6% vs 17.8%, *p* < .001). No differences in PFS were noted between patients who did and did not experience IRAEs based on a landmark PFS analysis. It is possible no differences in PFS were observed in patients based upon IRAE presence in the landmark analysis because the patients who progressed prior to 12 weeks were excluded. Although this information is not provided in the original manuscript, it is possible many early progressors did not experience IRAEs.

#### RCC

A two-center retrospective experience explored outcomes in metastatic RCC patients on first- or second-line treatment with ICIs based upon IRAE presence [17]. Of 90 patients treated with ICIs, 42.2% experienced IRAEs. The most common IRAEs were dermatologic (15.6%), gastrointestinal (14%) and endocrine (11%). In a multivariate analysis of IRAEs and a prognostic risk score (Heng), IRAEs were associated with improved OS (HR 0.38; 95% CI 0.18–0.79; *p* = .01) and time to next treatment (HR 0.48; 95% CI 0.28–0.83; *p* = .008).

A retrospective analysis of 389 pre-treated metastatic RCC patients who received therapy with nivolumab, was performed from an Italian RCC Early Access Program database [40]. One of the secondary endpoints of the study was assessing the association between IRAE onset and patient outcomes. In the included patients, 20% experienced any IRAEs with the most common sites being dermatologic (8%), gastrointestinal (5%) and endocrine (4%). Patients who experienced IRAEs had prolonged OS compared to those who did not experience them (NR vs 16.8 months, *p* = .002). In terms of 1-year OS, 1-year OS was 75.4 and 59.8% in patients who did and did not experience IRAEs, respectively.

#### UCC

A pooled analysis of 7 trials, including 1747 cisplatin-ineligible and cisplatin-refractory patients, was recently published [20]. All patients included in the analysis were treated with atezolizumab or pembrolizumab. The primary outcome of the analysis was to assess the relationship between patient outcome and development of IRAEs or adverse events of special interest (AESI). AESI were defined separately from IRAEs as autoimmune toxicities which did not require corticosteroid management. Using logistic regression, the odds ratio (OR) of experiencing an AESI was 5.38 in responders compared to non-responders; the OR of experiencing an IRAE was 3.77. Results from a responder analysis of the relationship between AESI or IRAE development and OS, when adjusted for baseline covariates, found an improvement in OS among patients who developed an AESI (HR 0.45; 95% CI 0.39–.53) or IRAE (HR 0.53; 95% CI 0.43–0.66). Responding patients who did or did not receive systemic corticosteroids seemed to have similar response duration (HR 1.09; 95% CI 0.7–1.69).

A single-center retrospective analysis in metastatic UCC patients also assessed outcomes in patients based on IRAE presence [41]. Of 52 included platinum-pretreated or -ineligible patients treated with anti-PD-1 or anti-PD-L1 antibodies, IRAEs were observed in 57.7% of patients. The most frequent grade 3/4 IRAE sites in these patients were gastrointestinal (13.2%) and dermatologic (6.6%). DCR (79% vs 36.3%, *p* = .002) and OS (21.91 vs 6.47 months, *p* = .004) were higher in patients with IRAEs compared to those without them.

#### Gastrointestinal

A retrospective analysis explored the relationship between IRAE onset and anti-PD-1 antibody efficacy in 61 gastrointestinal cancer patients (45.9% HCC, 44.2% MSI-H colorectal cancer and 9.8% GA & GEJ) with FDA-approved indications to receive ICIs [42]. Of included patients, 39.3% experienced IRAEs with the most common sites being musculoskeletal (29.4%), dermatologic (26.5%) and endocrine (20.6%). Patients who experienced IRAEs had a prolonged PFS and OS compared to those who did not (PFS: 32.4 vs 4.8 months, *p* = .0001; OS: 32.4 vs 8.5 months, *p* = .0036). Pre-specified subgroup analyses explored PFS and OS among patients who experienced IRAEs by IRAE severity (grade 3/4 vs grade 1/2), management (steroidal vs non-steroidal) and timing of onset (< 6 weeks vs ≥ 6 weeks). No statistically significant differences in PFS and OS were found in patients who experienced IRAEs based upon IRAE severity, management and timing of onset.

Another retrospective analysis specifically explored the relationship between IRAE onset and outcomes in gastric cancer patients treated with nivolumab [43]. Of 65 patients, 21.5% developed IRAEs with the most common site of involvement being gastrointestinal (35.7%). Patients who experienced IRAEs had prolonged PFS (7.5 vs 1.4 months (HR .11, *p* < .001)) and OS (16.8 vs 3.2 months (HR .17, *p* < .001)) compared to patients who did not experience them.

#### Head and neck

In an analysis of 114 patients with metastatic HNSCC treated with anti-PD-1 antibodies, unselected for PD-L1 status, patient outcomes were compared in patients by the presence or absence of IRAEs in both univariate and multivariate analyses [44]. Of the patients, 43% experienced IRAEs with the most common sites being dermatologic (33.9%), musculoskeletal (25.4%) and endocrine (23.7%). Patients with IRAEs had improved ORR (30.6% vs 12.3%, *p* = .02), PFS (6.9 vs 2.1 months, *p* = .0004) and OS (12.5 vs 6.8 months, *p* = .0007) compared to those without IRAEs. On multivariate analyses, IRAE onset was independently associated with improved ORR (*p* = .03), PFS (*p* = .0009) and OS (*p* = .003).

Table 1 is a summary of the studies previously discussed and includes the outcomes compared between patients with and without IRAEs in each study.

**Table 1:** Studies Comparing Outcomes in Advanced Malignancy Patients on Treatment with Anti-Programmed Cell Death Protein 1 (PD-1) and Anti-Programmed Death-Ligand 1 (PD-L1) Antibodies

Study	Disease	Number of Patients	Checkpoint Inhibitor(s) Used	Survival Endpoints Between Patients With and Without IRAES	Response Endpoints Between Patients With and Without IRAEs
Grangeon et al. [18]	NSCLC	270	Anti-PD-1 and anti-PD-L1	OS (HR 0.29; 95% CI 0.18–0.46; p = .001), PFS (HR 0.42; 95% CI 0.32–0.57; *p* < .001)	ORR (22.9% vs 5.7%, *p* < .0001), DCR (76% vs 58%, *p* < .001)
Ricciuti et al. [34]	NSCLC	195	Nivolumab	OS (HR 0.33; 95% CI 0.23–0.47; *p* < .001), PFS (HR 0.41; 95% CI 0.3–0.57; *p* < .001)	ORR (43.5% vs 10%, *p* < .001), DCR (70.5% vs 18.1%, *p* < .0001)
Riudavets et al. [13]	NSCLC, UCC and melanoma	178	Nivolumab, pembrolizumab and atezolizumab	OS (37.3 vs 7.8 months, *p* < 0.0001), PFS (7.9 vs 2.6 months, *p* < 0.0001)	Not provided
Sato et al.^a^ [19]	NSCLC	38	Nivolumab	PFS (HR 0.1; 95% CI .02–.37; *p* < .001)	ORR (63.6% vs 7.4%, *p* < .01)
Weber et al. [38]	Melanoma	576	Nivolumab	PFS (no significant differences between either group; HR or *p* value not provided)	ORR (48.6% vs 17.8%, *p* < .001)
Indini et al. [37]	Melanoma	173	Anti-PD-1	OS (HR 0.39; 95% CI 0.18–0.81; *p* = .007), PFS (HR 0.47; 95% CI 0.26–0.86; *p* = .016)	ORR (HR 1.95; 95% CI 0.91–4.15; *p* < 0.082), DCR (HR 1.98; 95% CI 1.07–3.67; *p* < 0.029)
Elias et al. [17]	RCC	90	Anti-PD-1	OS (HR 0.38; 95% CI 0.18–0.79; p = .01) and TTNT (HR 0.48; 95% CI 0.28–0.83; *p* = .008)	Not provided
Verzoni et al. [39]	RCC	389	Nivolumab	OS (HR .57; 95% CI .35–.93; *p* = .02)	Not provided
Maher et al. [20]	UCC	1747	Atezolizumab or pembrolizumab	OS (HR 0.53; 95% CI 0.43–0.66)	Not provided
Morales-Berera et al. [40]	UCC	52	Anti-PD-1 or anti-PD-L1	OS (21.91 vs 6.47 months, *p* = .004)	DCR (79% vs 36.3%, *p* = .002)
Das et al. [41]	GI	61	Anti-PD-1 monotherapy or in combination	OS (32.4 vs 8.5 months, *p* = .0036), PFS (32.4 vs 4.8 months, *p* = .0001)	Not provided
Masuda et al. [42]	Gastric	65	Nivolumab	OS (HR .17, *p* < .001), PFS (HR .11, *p* < .001)	Not provided
Foster et al. [43]	HNSCC	114	Anti PD-1	OS (12.5 vs 6.8 months, *p* = .0007), PFS (6.9 vs 2.1 months, *p* = .0004)	ORR (30.6% vs 12.3%, *p* = .02)

*Abbreviations: NSCLC* non-small cell lung cancer, *UCC* urothelial cell carcinoma, *RCC* renal cell carcinoma, *GI* gastrointestinal, *HNSCC* head and neck squamous cell carcinoma, *IRAEs* immune related adverse events, *OS* overall survival, *PFS* progression-free survival, *ORR* overall response rate, *DCR* disease control rate, *HR* hazard ratio, *CI* confidence interval, *TTNT* time to next treatment, *vs* versus

^a^Prospective study

### Anti-CTLA-4 antibodies

The data exploring the association between anti-CTLA-4 antibody-induced IRAEs and ICI efficacy arises largely from patients with melanoma, with mixed results. Starting with the positive studies, in a prospective study of 56 progressive metastatic melanoma patients treated with ipilimumab 3 mg/kg every 3 weeks or 1 mg/kg every 3 weeks after the initial dose, patients who experienced grade 3/4 IRAEs had an improved ORR compared to those who did not experience grade 3/4 IRAEs (36% vs 5%, *p* = .008) [45]. Of patients who experienced IRAEs, the most commonly involved sites were gastrointestinal (50%) and dermatologic (28.5%). In another prospective effort, 139 patients with pre-treated metastatic melanoma were treated with ipilimumab 3 mg/kg every 3 weeks or 1 mg/kg every 3 weeks after the initial dose (with and without peptide vaccinations) [22]. Of the included patients, 62% experienced any grade IRAE. The most common IRAEs were dermatologic (47.6%) and musculoskeletal (10.4%). Among patients who did and did not experience IRAEs, ORR was 26 and 2%, respectively (*p* = .004).

In a retrospective analysis of 198 metastatic pre-treated melanoma patients who received ipilimumab 3 mg/kg every 3 weeks for 4 doses, 29.8% experienced IRAEs [21]. Among responding patients, a higher proportion experienced any grade IRAEs compared to no IRAEs (*p* = .04). Another retrospective analysis assessed SEER database outcomes in 858 melanoma patients older than 65 treated with ipilimumab. Of these patients, 20.7% experienced IRAEs with the most common sites of involvement being gastrointestinal (17.5%), endocrine (10.5%) and dermatologic (5.4%). Patients who experienced non-severe IRAEs, severe IRAEs and no IRAEs had OS of 1.1, 0.9 and 0.6 years, respectively (*p* < .001).

Moving to the studies which question the IRAE onset and ICI efficacy hypothesis, a retrospective single institution analysis of 298 melanoma patients treated with ipilimumab 3 mg/kg assessed the association between time to treatment failure and OS by IRAE presence [46]. Of the included patients, 85% experienced any IRAEs. The most common IRAEs involved gastrointestinal (63.4%) and dermatologic organ systems. No differences in time to treatment failure and OS were detected between patients who did and did not experience IRAEs by landmark analyses. This absence of difference was also maintained when patients were stratified by whether they received systemic corticosteroids for IRAE management. It is possible no difference was observed in patients based upon IRAE presence because of the schedule of ipilimumab administration. Among the treated patients, 91% received 4 doses of ipilimumab while only 9% received > than 4 doses. It is possible the limited exposure to ipilimumab was not a sufficient time period for the relationship between IRAEs and OS to manifest in treated patients.

A phase I/II study of 88 unresectable or metastatic melanoma patients assessed the safety and efficacy profile of ipilimumab [47]. An exploratory analysis assessed the relationship between IRAE presence and DCR. Of the patients in the analysis, 72% developed IRAEs. There was no significant association between IRAE presence and DCR (*p* = .45) however in a group in the cohort with the highest DCR rate (39%), grade 3/4 IRAEs were associated with improved DCR (*p* = .03).

An aggregate analysis of 3 phase II studies in metastatic melanoma patients treated with ipilimumab assessed the relationship between DCR and OS in patients with and without IRAEs [48]. Although DCR was higher in patients who experienced any IRAEs compared to those who did not experience them (rates not specified), there was no statistically significant difference in DCR between patients with grade 1 vs grade ≥ 2 IRAEs (20–24% vs 34%). OS was also improved in patients who experienced IRAEs compared to those who did not experience them (14.8 vs 8.2 months) however did not differ by grade in patients who experienced IRAEs.

### Nuances of the association between IRAEs and ICI efficacy

#### Bias

Before discussing how specific IRAE characteristics (site, severity, timing, management) may influence ICI efficacy, it is important to discuss time on therapy, a potential confounding factor in the relationship between IRAEs and ICI response. The notion that patients who experience IRAEs are those who remain on ICIs for longer time periods and thus have a better prognosis than those who do not, by virtue of their disease biology, could be a source of guarantee-time bias [49]. Adjuvant studies, therefore, with their low rates of on-treatment relapses, present one setting where this bias may be substantially mitigated [50]. In an adjuvant study of 1019 resected Stage IIIA, IIIB and IIIC melanoma patients treated with pembrolizumab or placebo, patients who experienced IRAEs in the treatment arm experienced prolonged relapsed-free survival (RFS) compared to those who did not (HR 0.61; 95% CI 0.39–0.95; *p* = .03). No association between IRAE onset and RFS was witnessed in the placebo arm. Compared to the placebo treated patients, the hazard of relapse or death was reduced in the pembrolizumab treated patients after IRAE onset (HR 0.37; 95% CI 0.24–0.57) than before IRAE onset (HR 0.61; 95% CI 0.49–0.77) (*p* = .028).

Another study which suggests time on therapy is not the reason for the relationship between IRAE onset and ICI efficacy was a pooled retrospective analysis of melanoma patients from the randomized Checkmate 067 and Checkmate 069 trials [51]. In this analysis, 409 treatment naïve unresectable melanoma patients received induction therapy with nivolumab 1 mg/kg plus ipilimumab 3 mg/kg every 3 weeks for 4 doses prior to being transitioned to nivolumab 3 mg/kg every 2 weeks thereafter. Of these patients, 176 (43%) discontinued treatment due to IRAEs (classified in the analysis as treatment related AEs). Median duration of treatment was 1.4 months and 9.4 months in patients who discontinued the ICIs due to IRAEs in the induction phase and those who did not discontinue treatment due to IRAEs, respectively. ORR was 58.3 and 50.2% in patients who did and did not discontinue ICIs during the induction phase, respectively (*p* = .18). No difference in PFS (HR .99; 95% CI .72–1.34; *p* = .97) or OS (HR .79; 95% CI .54–1.17; *p* = .23) was observed between patients who did and did not discontinue ICIs during the induction phase. The findings from this analysis suggest IRAE onset may be more predictive of ICI response than time on therapy, as patients who had to discontinue therapy due to IRAEs (with markedly less time on the drugs) had similar ORR, PFS and OS compared to patients who remained on therapy.

#### Site

Several of the previously mentioned studies suggest dermatologic and endocrine IRAEs are associated with ICI response. In this section we will discuss other studies which lead credence to this notion. A retrospective analysis of 83 metastatic cancer patients (66 of whom had melanoma) treated with pembrolizumab explored the association between cutaneous IRAEs and treatment efficacy [52]. Of these patients, 42% experienced cutaneous IRAEs. Patients who experienced cutaneous IRAEs, at any dose of pembrolizumab, had a significantly longer PFS than those who did not (*p* < .001; *p* < .04; *p* < .007).

A 318-patient single-center retrospective analysis examined the relationship between dermatologic IRAEs and ICI efficacy in advanced melanoma patients [53]. Patients in the analysis were treated with anti-PD-1 antibody monotherapy or in combination with ipilimumab. Among patients who developed dermatologic IRAEs, RR (60% vs 27%, *p* < .001), PFS (797 vs 112 days, *p* < .001) and OS (1691 vs 526 days, *p* < .001) were all superior to these measures in patients who did not develop dermatologic IRAEs. Multivariate logistic regression, controlling for age, combination therapy, prior therapy and sex, confirmed an independent association of dermatologic IRAEs with superior RR (OR 3.58; 95% CI 2.17–5.90; *p* < .001). In addition, numerous studies have suggested that vitiligo, while relatively uncommon with anti-PD-1 therapy (although up to 10% in melanoma patients), is associated with extremely high response rates (70–80%) across immunotherapies.

A systematic literature review pooling 12 RCTs identified 3815 metastatic head & neck and lung cancer patients treated with ICIs (unspecified distribution of anti-PD-1 and anti-CTLA-4 antibodies) [54]. The primary aim of the analysis was to assess the prevalence of endocrine IRAEs and the association between endocrine IRAEs and patient outcomes. The most common endocrine IRAE reported was hypothyroidism and a significant correlation between endocrine IRAEs and OS was observed (*p* = .019).

A recent publication cited above suggests IRAE sites associated with ICI efficacy may have more to do with shared antigens between tumor and involved site rather than any intrinsic association between checkpoint inhibitor and IRAE site [30]. Further investigation is needed to clarify whether certain IRAE sites are predictive of ICI response or whether organ specific IRAEs result strictly from shared antigens between site and tumor.

#### Severity

IRAEs are thought to represent bystander effects from activated T-cells and as such, mechanistically, patients who experience more severe IRAEs should have increased T-cell activity and experience better outcomes compared to those who experience lower grade IRAEs [27]. Most of the previously discussed studies with anti-PD-1 and anti-CTLA-4 antibodies do not demonstrate any relationship between IRAE severity and ICI efficacy. This could be explained by the fact that patients with severe IRAEs tend to experience significant morbidity and sometimes mortality from the autoimmune reactions which muddles the difference in survival between patients with and without IRAEs [53]. Further, severe toxicity is often associated with more aggressive immunosuppression, which may also influence efficacy (see management).

#### Timing

The implications of timing of IRAE onset and ICI efficacy has been much less studied. Previously referenced studies in NSCLC and gastrointestinal cancer patients have not demonstrated a relationship between earlier IRAE onset and increased ICI response. A study in melanoma patients also did not demonstrate this relationship [55]. In a retrospective analysis of metastatic melanoma patients receiving combination therapy with anti-PD-1 and anti-CTLA-4 antibodies, 80 patients experienced IRAEs within 21 days. Among these patients who developed rapid IRAEs, RR was 54% and median PFS was 8.74 months, which was in line with outcomes seen in patients on trial treated with the combination.

Several studies, however, suggest an association between timing of IRAE onset and ICI benefit, although this is not uniformly maintained across outcomes. A prospective study in 43 metastatic NSCLC lung cancer patients treated with nivolumab assessed PFS, ORR and DCR between patients who experienced IRAE onset at ≤2 and ≤ 6 weeks [36]. Both ORR and DCR were higher in patients who experienced IRAEs at ≤2 weeks and ≤ 6 weeks compared to those who did not experience them. This same trend held true for PFS although only reached statistical significance in the ≤2 weeks IRAE onset cohort. However, extremely late toxicities are typically only observed in patients benefiting from treatment, as described above.

A retrospective analysis analyzed aggregate data from two phase I studies of durvalumab and durvalumab plus tremelimumab across solid tumor types and assessed whether timing of IRAE onset was associated with RR and OS [56]. Patients who experienced ≥1 IRAE has statistically significant improvements in OS compared to those who did not experience IRAEs at weeks 4, 8, 12, 16, 20 and 24 in both studies. RR was also improved in patients who experienced IRAEs compared to those who did not at weeks 12, 16, 20 and 24 in both studies.

A single-center analysis assessed whether metastatic UCC patients who developed IRAEs demonstrated clinical benefit based on timing of onset [57]. Of 199 total patients treated with anti-PD-1 and anti-PD-L1 antibodies, in patients who developed IRAE onset < 90 days, DCR was 40.6% compared to 17.8% patients who did not develop IRAEs (*p* = .008). No statistically significant differences were observed for either 6-month PFS (35.3% vs 19.2%, *p* = .21) or 1-year OS (57.7% vs 41.8%, *p* = .18) between patients who developed IRAE onset < 90 days and in those who did not develop IRAEs.

#### Management

Nearly all the previously referenced studies, which assessed the impact of corticosteroids for IRAE management on ICI outcomes, did not demonstrate worse outcomes in patients requiring corticosteroids. However, several studies have questioned this assumption. One small study of melanoma patients who developed hypophysitis while on treatment with ipilimumab, revealed patients who received lower dose corticosteroids had substantially better survival compared with those treated with high-dose corticosteroids [58]. Another study in NSCLC patients treated with anti-PD-1 and anti-PD-L1 antibodies, suggested that patients receiving corticosteroids at baseline (when treatment is initiated) fare worse than those not on corticosteroids [59]. One could speculate that although toxicity is associated with superior outcomes, this association is partially blunted by high-dose corticosteroids. Large series comparing patients treated with distinct doses of corticosteroids are needed to help sort this out; such analyses are ongoing. However, corticosteroids (higher than physiologic doses) used while initiating therapy do appear to dampen therapeutic responses.

### Efforts to uncouple autoimmunity from anti-tumor effect

Although IRAE onset appears to be linked with ICI response, it is unclear whether this is an inevitable association, and efforts are ongoing to uncouple response from toxicity. One proof of principle study was a phase II trial combining high-dose ipilimumab with or without sargramostim (GM-CSF) in metastatic melanoma patients [60]. Patients treated with GM-CSF exhibited lower toxicities and improved survival compared to patients treated with high-dose ipilimumab alone; response rates were equivocal in the two arms. Although the mechanism of this effect is not clear, a phase III study of ipilimumab and nivolumab with or without GM-CSF is ongoing (NCT02339571). IL-6 is a cytokine which may represent another such target. A recent study analyzed RNA from patient-matched normal colonic tissue and IRAE-induced colitis tissue [61]. Differences in gene expression from normal and colitis tissue, along with baseline and on-treatment tumor biopsies from responding versus non-responding patients to ipilimumab, were analyzed. In tissue from patients with IRAE-induced colitis, the gene with the greatest degree of differential upregulation from normal colonic tissue was IL-6. IL-6, along with other differentially upregulated genes in colitis tissue from patients, was not significantly upregulated in responding tumors. Interestingly, IL-6 was also the gene which was differentially upregulated in tumor tissue from non-responding patients. The investigators blocked IL-6 in combination with CTLA-4 in mouse models which created significant tumor shrinkage beyond that seen in mice treated with anti-CTLA-4 antibodies alone. Anti-IL-6 directed therapy in combination with ICIs has not yet been tested clinically. A clinical trial in metastatic melanoma patients, which has since been terminated, combined nivolumab plus ipilimumab with the alpha 4 beta 7 integrin antagonist antibody vedolizumab and the human chemokine receptor 2 antagonist antibody plozalizumab in order to clinically demonstrate the potential for uncoupling anti-tumor activity and autoimmunity [NCT02723006]. Findings from the patients treated on this study have not yet been reported.

## Conclusion

There appears to be an intimate link between autoimmunity and anti-tumor effect elicited by ICIs. An emerging area of research interest in the field of oncology is whether these two aspects of ICIs can be uncoupled to maximize benefit while minimizing toxicities for patients. IRAEs appear to represent a clinical biomarker for ICI response, albeit one that emerges on treatment. Within ICIs, IRAE onset appears to be more strongly associated with anti-PD-1 and anti-PD-L1 antibody response than response to anti-CTLA-4. This perhaps may be more a by-product of the diseases for which each of the agents are FDA-approved, the differential mechanisms of action between the agents or the time-course of treatment (e.g. 4 doses then discontinuation for anti-CTLA-4 vs long-term treatment for anti-PD-1 or anti-PD-L1). Many questions remain about the true nature of the relationship between IRAE characteristics such as site, severity, timing of onset and management and ICI efficacy. Prospective well-powered studies need to be performed to understand the true implications of IRAE characteristics on ICI response in patients.

## Data Availability

Data sharing is not applicable to this article as no datasets were generated or analyzed during the current study.

## Abbreviations

CI: Confidence interval
CTLA-4: Cytotoxic T-lymphocyte-associated protein 4
DCR: Disease control rate
GA & GEJ: Gastric and gastroesophageal junction
HNSCC: Head and neck squamous cell carcinoma
HR: Hazard ratio
ICIs: Immune checkpoint inhibitors
IRAE: immune-related adverse event
MSI-H: Microsatellite instability-high
NSCLC: Non-small cell lung cancer
OR: Odds ratio
ORR: Overall response rate
OS: Overall survival
PD-1: Programmed cell death protein 1
PD-1: Programmed death-ligand 1
PFS: Progression-free survival
RCC: Renal cell carcinoma
RFS: Relapse-free survival
TCR: T-cell receptor
UCC: Urothelial carcinoma
vs: versus

## References

[1] Robert C, Long G, Brady B, Dutriaux C, Maio M, Mortier M (2015). Nivolumab in previously untreated melanoma without BRAF mutation. N Engl J Med.

[2] Robert C, Schachter J, Long G, Arance A, Grob JJ, Mortier L (2015). Pembrolizumab versus Ipilimumab in Advanced Melanoma. N Engl J Med.

[3] Motzer R, Escudier B, McDermott D, George S, Hammers HJ, Srinivas S (2015). Nivolumab versus Everolimus in advanced renal-cell carcinoma. N Engl J Med.

[4] Bellmunt J, de Wit R, Vaughn DJ, Fradet Y, Lee J, Fong L (2017). Pembrolizumab as second-line therapy for advanced Urothelial carcinoma. N Engl J Med.

[5] Ferris R, Blumenschein G, Fayette J, Guigay J, Colevas AD, Licitra L (2016). Nivolumab for recurrent squamous-cell carcinoma of the head and neck. N Engl J Med.

[6] Reck M, Rodriguez-Abreu D, Robinson AG, Hui R, Csoszi T, Fulop A (2016). Pembrolizumab versus chemotherapy for PD-L1–positive non–small-cell lung Cancer. N Engl J Med.

[7] Le D, Durham J, Smith K, Wang H, Bartlett BR, Aulakh LK (2017). Mismatch repair deficiency predicts response of solid tumors to PD-1 blockade. Science..

[8] Overman M, McDermott R, Leach JL, Lonardi S, Lenz HJ, Morse MA (2017). Nivolumab in patients with metastatic DNA mismatch repair-deficient or microsatellite instability-high colorectal cancer (CheckMate 142): an open-label, multicentre, phase 2 study. Lancet Oncol.

[9] El-Khoueiry A, Sangro B, Yau T, Crocenzi CS, Kudo M, Hsu C (2017). Nivolumab in patients with advanced hepatocellular carcinoma (CheckMate 040): an open-label, non-comparative, phase 1/2 dose escalation and expansion trial. Lancet..

[10] Fuchs CS, Doi T, Jang RW, Muro K, Satoh T, Machado M (2018). Safety and efficacy of Pembrolizumab Monotherapy in patients with previously treated advanced gastric and Gastroesophageal junction Cancer: phase 2 clinical KEYNOTE-059 trial. JAMA Oncol.

[11] Mouw K, Goldberg M, Konstantinipoulos P, D’Andrea A (2017). DNA damage and repair biomarkers of immunotherapy response. Cancer Discov.

[12] Goodman A, Kato S, Bazhenova L, Patel SP, Frampton GM, Miller V (2017). Tumor mutational burden as an independent predictor of response to immunotherapy in diverse cancers. Mol Cancer Ther.

[13] Riudavets M, Barba A, Maroto P, Sullivan IG, Anguera G, Paez D (2018). Correlation between immune-related adverse events (irAEs) and efficacy in patients with solid tumors treated with immune-checkpoints inhibitors (ICIs). J Clin Oncol.

[14] Rogado J, Sanchez-Torres JM, Romero-Laorden N, Ballesteros AI, Pacheco-Barcia V, Ramos-Levi A (2019). Immune-related adverse events predict the therapeutic efficacy of anti–PD-1 antibodies in cancer patients. Eur J Cancer.

[15] Toi Y, Sugawara S, Kawashima Y, Aiba T, Kawana S, Saito R (2018). Association of Immune-Related Adverse Events with clinical benefit in patients with advanced non-small-cell lung Cancer treated with Nivolumab. Oncologist.

[16] Okada N, Kawazoe H, Takechi K, Matsudate Y, Utsunomiya R, Zamami Y (2019). Association between immune-related adverse events and clinical efficacy in patients with melanoma treated with Nivolumab: a multicenter retrospective study. Clin Ther.

[17] Elias R, Yan N, Singla N, Levonyack N, Formella J, Christie A (2019). Immune-related adverse events are associated with improved outcomes in ICI-treated renal cell carcinoma patients. J Clin Oncol.

[18] Grangeon M, Tomasini P, Chaleat S, Jeanson A, Souquet-Bressand M, Khobta N (2018). Association Between Immune-related Adverse Events and Efficacy of Immune Checkpoint Inhibitors in Non-small-cell Lung Cancer. Clin Lung Cancer.

[19] Sato K, Akamatsu H, Murakami E, Sasaki S, Kanai K, Hayata A (2018). Correlation between immune-related adverse events and efficacy in non-small cell lung cancer treated with nivolumab. Lung Cancer.

[20] Maher V. Ellen, Fernandes Laura L., Weinstock Chana, Tang Shenghui, Agarwal Sundeep, Brave Michael, Ning Yang-min, Singh Harpreet, Suzman Daniel, Xu James, Goldberg Kirsten B., Sridhara Rajeshwari, Ibrahim Amna, Theoret Marc, Beaver Julia A., Pazdur Richard (2019). Analysis of the Association Between Adverse Events and Outcome in Patients Receiving a Programmed Death Protein 1 or Programmed Death Ligand 1 Antibody. Journal of Clinical Oncology.

[21] Eigentler TK, Schlaak M, Hassel JC, Loquai C, Stoffels I, Gutzmer R (2014). Effectiveness and tolerability of ipilimumab: experiences from 198 patients included in a named-patient program in various daily-practice settings and multiple institutions. J Immunother.

[22] Downey SG, Klapper JA, Smith FO, Yang JC, Sherry RM, Royal RE (2007). Prognostic factors related to clinical response in patients with metastatic melanoma treated by CTL-associated antigen-4 blockade. Clin Cancer Res.

[23] Weber JS, O'Day S, Urba W, Powderly J, Nichol G (2008). Yellin met, et al. phase I/II study of ipilimumab for patients with metastatic melanoma. J Clin Oncol.

[24] Mian I, Yang M, Zhao M, Shah M, Diab A, Shannon V (2016). Immune-related adverse events and survival in elderly patients with melanoma treated with ipilimumab. J Clin Oncol.

[25] Ribas A, Camacho LH, Lopez-Berestein G, Pavlov D, Bulanhagui CA, Millham R (2005). Antitumor activity in melanoma and anti-self responses in a phase I trial with the anti-cytotoxic T lymphocyte-associated antigen 4 monoclonal antibody CP-675,206. J Clin Oncol.

[26] Yoest J (2017). Clinical features, predictive correlates, and pathophysiology of immune-related adverse events in immune checkpoint inhibitor treatments in cancer: a short review. Immunotargets Ther.

[27] Passat T, Touchefeu Y, Gervois N, Jarry A, Bossard C, Bennouna J (2018). Physiopathological mechanisms of immune-related adverse events induced by anti-CTLA-4, anti-PD-1 and anti-PD-L1 antibodies in cancer treatment. Bull Cancer.

[28] Johnson DB, Balko JM, Compton ML, Chalkias S, Gorham J, Xu Y (2016). Fulminant myocarditis with combination immune checkpoint blockade. N Engl J Med.

[29] Berner Fiamma, Bomze David, Diem Stefan, Ali Omar Hasan, Fässler Mirjam, Ring Sandra, Niederer Rebekka, Ackermann Christoph J., Baumgaertner Petra, Pikor Natalia, Cruz Cristina Gil, van de Veen Willem, Akdis Mübeccel, Nikolaev Sergey, Läubli Heinz, Zippelius Alfred, Hartmann Fabienne, Cheng Hung-Wei, Hönger Gideon, Recher Mike, Goldman Jonathan, Cozzio Antonio, Früh Martin, Neefjes Jacques, Driessen Christoph, Ludewig Burkhard, Hegazy Ahmed N., Jochum Wolfram, Speiser Daniel E., Flatz Lukas (2019). Association of Checkpoint Inhibitor–Induced Toxic Effects With Shared Cancer and Tissue Antigens in Non–Small Cell Lung Cancer. JAMA Oncology.

[30] Gopalakrishnan V, Spencer CN, Nezi L, Reuben A, Andrews MC, Karpinets TV (2018). Gut microbiome modulates response to anti-PD-1 immunotherapy in melanoma patients. Science..

[31] Matson V, Fessler J, Bao R, Chongsuwat T, Zha Y, Alegre M (2018). The commensal microbiome is associated with anti–PD-1 efficacy in metastatic melanoma patients. Science..

[32] Chaput N, Lepage P, Coutzac C, Soularue E, Le Roux K, Monot C (2017). Baseline gut microbiota predicts clinical response and colitis in metastatic melanoma patients treated with ipilimumab. Ann Oncol.

[33] Iwama S, De Remigis A, Callahan MK, Slovin SF, Wolchok JD, Caturegli P (2014). Pituitary Expression of CTLA-4 Mediates Hypophysitis Secondary to Administration of CTLA-4 Blocking Antibody. Sci Transl Med.

[34] Ricciuti B, Genova C, De Giglio A, Bassanelli M, Dal Bello MG, Metro G (2019). Impact of immune-related adverse events on survival in patients with advanced non-small cell lung cancer treated with nivolumab: long-term outcomes from a multi-institutional analysis. J Cancer Res Clin Oncol.

[35] Moor RJ, Roberts KM, Mason R, Gunawan B, Feng S, Hong JH (2018). Immune-related adverse events and nivolumab outcomes in non-small cell lung cancer patients: a multi-institutional, retrospective cohort study. J Clin Oncol.

[36] Teraoka S, Fujimoto D, Morimoto T, Kawachi H, Ito M, Sato Y (2017). Early immune-related adverse events and association with outcome in advanced non-small cell lung Cancer patients treated with Nivolumab: a prospective cohort study. J Thorac Oncol.

[37] Indini A, Di Guardo L, Cimminiello C, Prisciandaro M, Randon G, De Braud F (2019). Immune-related adverse events correlate with improved survival in patients undergoing anti-PD1 immunotherapy for metastatic melanoma. J Cancer Res Clin Oncol.

[38] Borcoman E, Nandikolla A, Long G (2018). Patterns of Response and Progression to Immunotherapy. Am Soc Clin Oncol Educ Book.

[39] Weber JS, Hodi FS, Wolchok JD, Topalian SL, Schadendorf D, Larkin J (2017). Safety profile of Nivolumab Monotherapy: a pooled analysis of patients with advanced melanoma. J Clin Oncol.

[40] Verzoni E, Cartenì G, Cortesi E, Giannarelli D, De Giglio A, Sabbatini R (2019). Real-world efficacy and safety of nivolumab in previously-treated metastatic renal cell carcinoma, and association between immune-related adverse events and survival: the Italian expanded access program. J Immunother Cancer.

[41] Morales-Barera R, Rodriguez CS, Gonzalez M, Ros J, Semidey ME (2019). Hernandez ES, et al. J Clin Oncol.

[42] Das S, Ciombor KK, Haraldsdottir S, Pumpalova Y, Sahin IH, Shyr Y (2019). Immune checkpoint inhibitors (ICIs) in gastrointestinal (GI) cancer: Immune-related adverse events (IRAEs) and efficacy. J Clin Oncol.

[43] Masuda K, Shoji H, Nagashima K, Yamamoto S, Ishikawa M, Imazeki H (2019). Correlation between immune-related adverse events and prognosis in patients with gastric cancer treated with nivolumab. J Clin Oncol.

[44] Foster CC, Kochanny S, Khattri A, Acharya R, Dekker A, Tan YC (2018). Association of immune-related adverse events (irAEs) with improved response, progression-free survival, and overall survival for patients with metastatic head and neck cancer receiving anti-PD-1 therapy. J Clin Oncol.

[45] Attia P, Phan GQ, Maker AV, Robinson MR, Quezado MM, Yang JC (2005). Autoimmunity correlates with tumor regression in patients with metastatic melanoma treated with anti-cytotoxic T-lymphocyte antigen-4. J Clin Oncol.

[46] Horvat TZ, Adel NG, Momtaz P, Postow MA, Callahan MK, Dang TO (2015). Immune-related adverse events, need for systemic immunosuppression, and effects on survival and time to treatment failure in patients with melanoma treated with Ipilimumab at memorial Sloan Kettering Cancer center. J Clin Oncol.

[47] Weber JS, O'Day S, Urba W, Powderly J, Nichol G, Yellin M (2008). Phase I/II study of ipilimumab for patients with metastatic melanoma. J Clin Oncol.

[48] Lutzky J, Wolchok J, Hamid O, Lebbe C, Pehamberger H, Linette G (2009). Association between immune-related adverse events (irAEs) and disease control or overall survival in patients (pts) with advanced melanoma treated with 10 mg/kg ipilimumab in three phase II clinical trials. J Clin Oncol.

[49] Giobbie-Hurder A, Gelber RD, Regan MM (2013). Challenges of guarantee-time bias. J Clin Oncol.

[50] Eggermont AM, Kicinski M, Blank CU, Mandala M, Long G, Atkinson V (2019). Prognostic and predictive value of an immune-related adverse event among stage III melanoma patients included in the EORTC 1325/KEYNOTE-054 pembrolizumab versus placebo trial. J Clin Oncol.

[51] Schadendorf D, Wolchok J, Hodi FS, Chiarion-Sileni V, Gonzalez R, Rutkowski P (2017). Efficacy and safety outcomes in patients with advanced melanoma who discontinued treatment with Nivolumab and Ipilimumab because of adverse events: a pooled analysis of randomized phase II and III trials. J Clin Oncol.

[52] Sanlorenzo M, Vujic I, Daud A, Algazi A, Gubens M, Luna SA (2015). Pembrolizumab cutaneous adverse events and their association with disease progression. JAMA Dermatol.

[53] Quach HT, Dewan AK, Davis EJ, Ancell KA, Fan R, Ye F (2019). Association of Anti–Programmed Cell Death 1 cutaneous toxic effects with outcomes in patients with advanced melanoma. JAMA Oncol..

[54] Gomes-Lima CJ, Kwagyan J, King F, Fernandez SJ, Burman KD, Veytsman I (2019). A comprehensive meta-analysis of endocrine immune-related adverse events of immune checkpoint inhibitors and outcomes in head and neck cancer and lung cancer. J Clin Oncol.

[55] Dearden HC, Au L, Ying Wang D, Zimmer L, Eroglu Z, Smith JL (2018). Hyperacute toxicity with combination ipilimumab (ipi) and anti-PD1 immunotherapy. J Clin Oncol.

[56] Morehouse C, Abdullah SE, Dar M, Ranade K, Higgs B (2019). Early incidence of immune-related adverse events (irAEs) predicts efficacy in patients (pts) with solid tumors treated with immune-checkpoint inhibitors (ICIs). J Clin Oncol.

[57] Vitale NV, Pond GR, Alaiwi SA, Nassar A, Flippot R, Choueiri TK (2019). Association of immune-related adverse events (irAEs) with clinical benefit in patients with metastatic urothelial carcinoma (mUC) treated with immune-checkpoint inhibitors (ICIs). J Clin Oncol.

[58] Faje AT, Lawrence D, Flaherty K, Freedman C, Fadden R, Rubin K (2018). High-dose glucocorticoids for the treatment of ipilimumab-induced hypophysitis is associated with reduced survival in patients with melanoma. Cancer..

[59] Arbour KC, Mezquita L, Long N, Rizvi H, Auclin E, Ni A (2018). Impact of baseline steroids on efficacy of programmed cell Death-1 and programmed death-ligand 1 blockade in patients with non-small-cell lung Cancer. J Clin Oncol.

[60] Hodi FS, Lee S, McDermott D, Rao UN, Butterfield LH, Tarhini AA (2014). Sargramostim plus Ipilimumab vs Ipilimumab alone for treatment of metastatic melanoma: a randomized clinical trial. JAMA..

[61] Johnson DH, Hailemichael Y, Foo WC, Hess KR, Haymaker CL, Wani KM (2019). Interleukin-6 is potential target to de-couple checkpoint inhibitor-induced colitis from antitumor immunity. J Clin Oncol.

